# Correction: Integrative Modeling of eQTLs and Cis-Regulatory Elements Suggests Mechanisms Underlying Cell Type Specificity of eQTLs

**DOI:** 10.1371/journal.pgen.1004546

**Published:** 2014-07-03

**Authors:** The PLOS Genetics Staff

In Table S2, column #13 (‘BETA') is incorrect. A corrected version of Table S2 can be viewed below.

## Supporting Information

Table S2eQTL SNP replication table. Summary of eQTL findings and replication across all included studies. eQTL SNPs were included in the table if, from a given study, they were the most highly associated cis-linked SNP within an LD block and if their 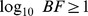. eQTL SNPs are listed in separate rows and associated data are in columns, as labeled in the first row and denote the following:1. REF STUDY. eQTL discovery study.2. GENE. RefSeq gene identifier.3. CHR. Chromosome of the eQTL SNP and associated gene expression trait.4. STRAND. Annotated strand of the associated transcript.5. TSS. Genomic coordinate of the transcription start site.6. TES. Genomic coordinate of the transcription end site.7. RSID. Identity of the eQTL SNP.8. HS INDEX. Identity of the LD block containing the SNP.9. RSCOORD. Genomic coordinate of the SNP.10. TIER. Tier of the SNP with respect the associated gene.11. UBF. Univariate 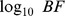 of the eQTL SNP-gene expression trait association.12. MBF. Multivariate 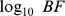 of the eQTL SNP-gene expression trait association, controlling for all SNPs in lower tiers.13. BETA. Estimate of the effect size per minor allele of the SNP.14. GEX. Mean gene expression level across all samples in the discovery study.15. PROBE MAF. Maximum minor allele frequency of SNPs overlapping the coordinates of the gene expression probe.16. NALN. Number of high quality alignments between the gene expression probe and hg18.17. STL, MBR, CLI, CPL, HVC, GCT, GCF, HCE, HPC, MLI, GCL. Each subsequent column denotes the measure of univariate replication between the SNP and gene expression trait in the study set indicated by the TLA, where:
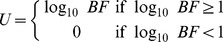

Click here for additional data file.
