# Supplementary figures and images for: Integrative Modeling of eQTLs and Cis-Regulatory Elements Suggests Mechanisms Underlying Cell Type Specificity of eQTLs
Source: PLoS Genet. 2013 Aug 1;9(8):e1003649. doi: 10.1371/journal.pgen.1003649 (PMC3731231; doi:10.1371/journal.pgen.1003649)

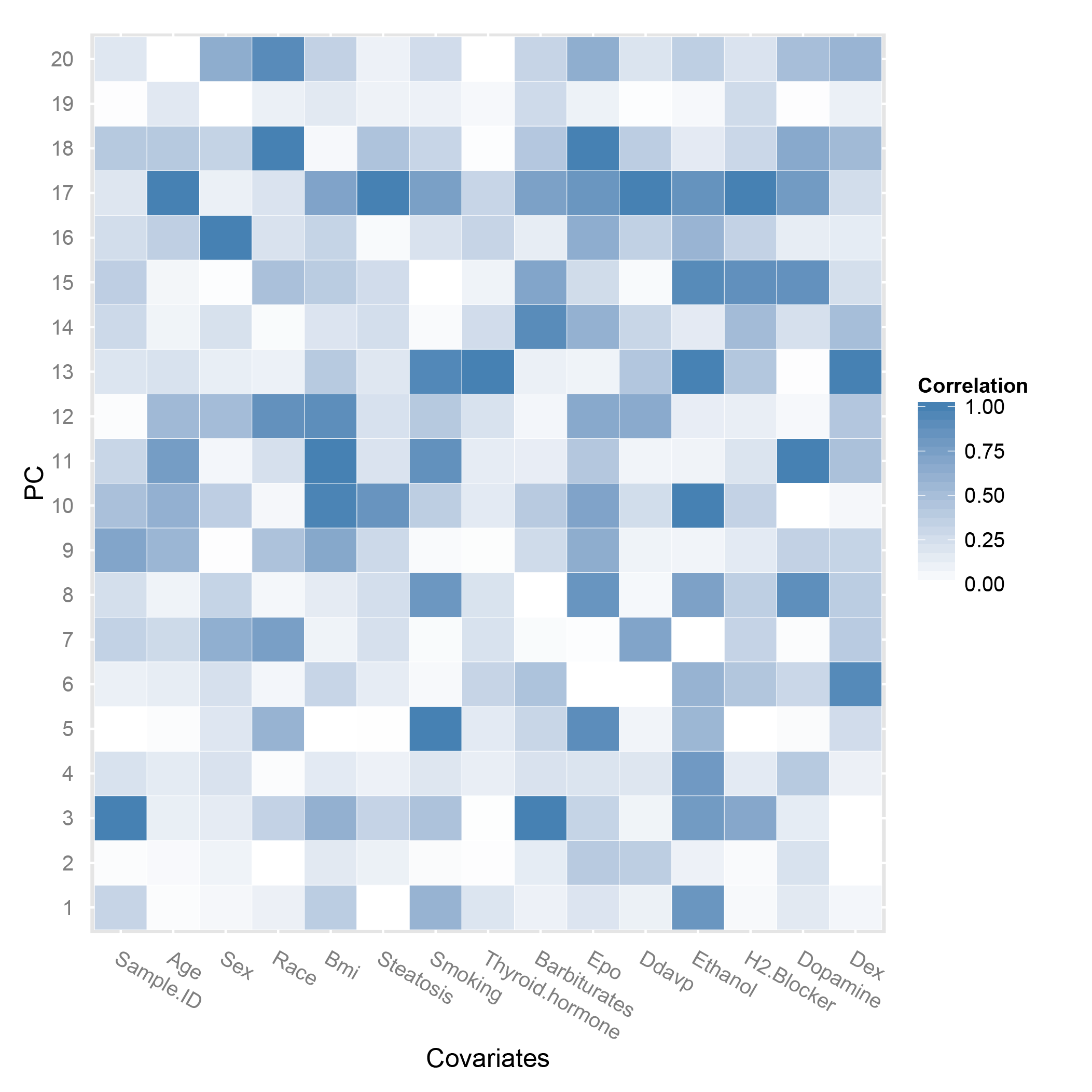

Supplement: Figure S1 — Sample principal components (PCs) capture many known study covariates in UChicago_liver study. Heat map indicating the absolute Pearson's correlation between sample covariates (x-axis) and the first twenty PCs (y-axis). Age, for example, is captured well by the PC and smoking status is captured best by the PC. Sample ID appears correlated with the PC because the IDs were ordered by when the sample was processed, which is well correlated with batch and other known gene expression confounders. (TIF) [file pgen.1003649.s001.tif]

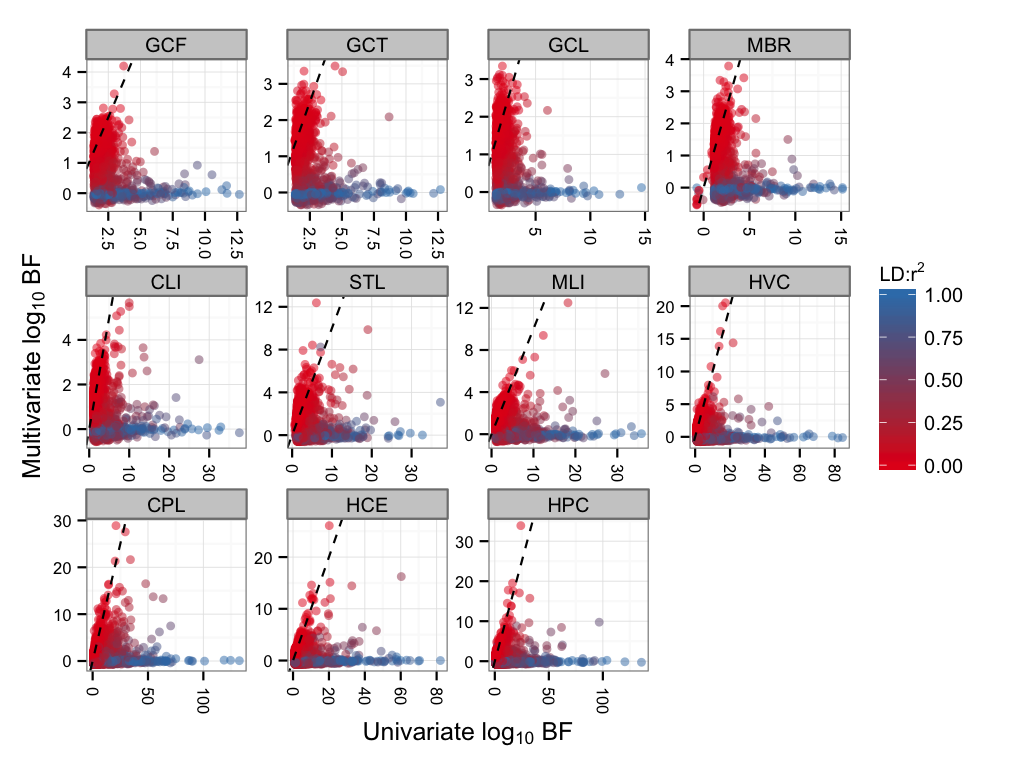

Supplement: Figure S2 — Identification of allelic heterogeneity. Univariate (x-axis) versus multivariate (y-axis) as a function of linkage disequilibrium (; color as indicated in scale bar) between the primary and secondary SNP. All tier two SNPs are plotted for each study independently, in each panel, as labeled at top. Note SNPs in higher LD with the primary SNP (‘bluer’ points) tend to produce greater drops from the univariate . (TIF) [file pgen.1003649.s002.tif]

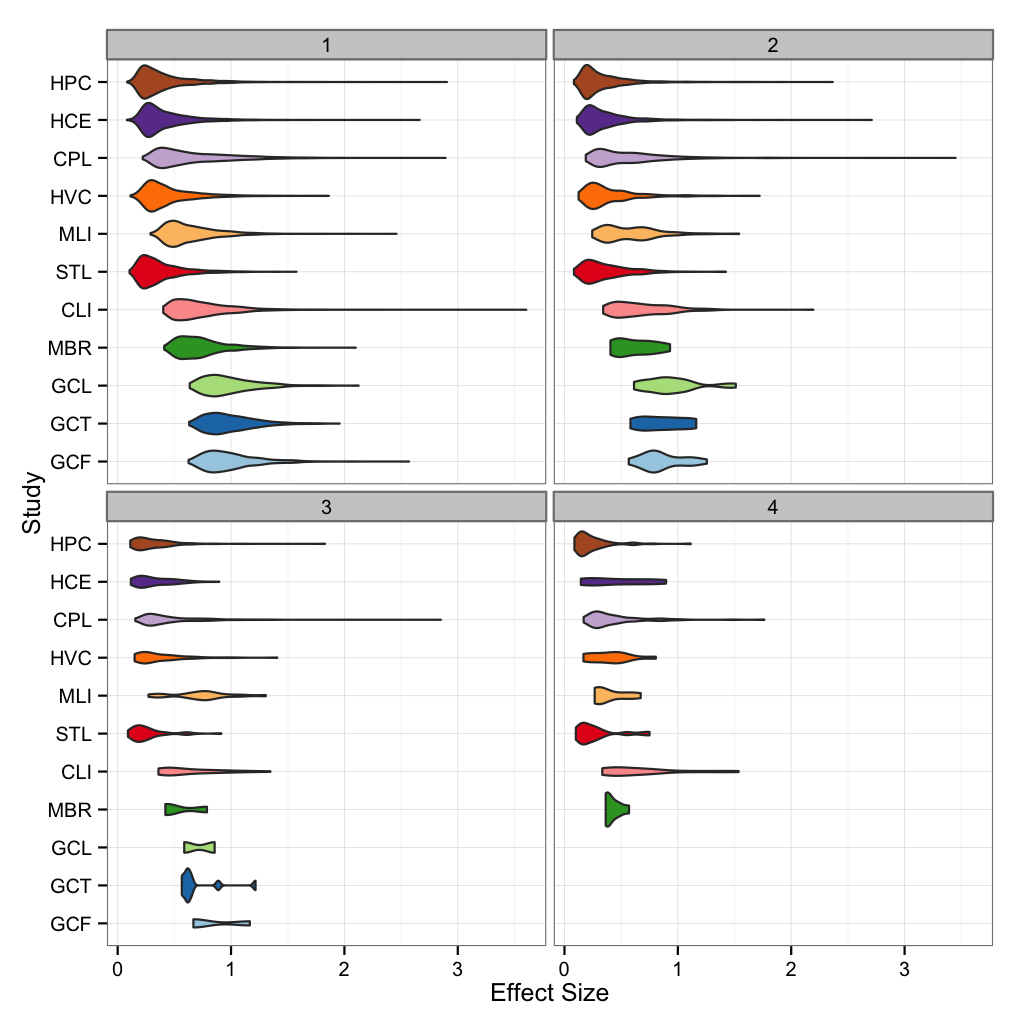

Supplement: Figure S3 — Distribution of eQTL effect sizes by study and SNP tier. Violin plots of the distribution of effect sizes for significant () eQTLs. Data are plotted separately for each study, as indicated by plot color and sample labels at left. SNPs from each tier are plotted separately in each facet, as labeled at top. Note studies are ordered on the y-axis by sample size. (TIF) [file pgen.1003649.s003.tif]

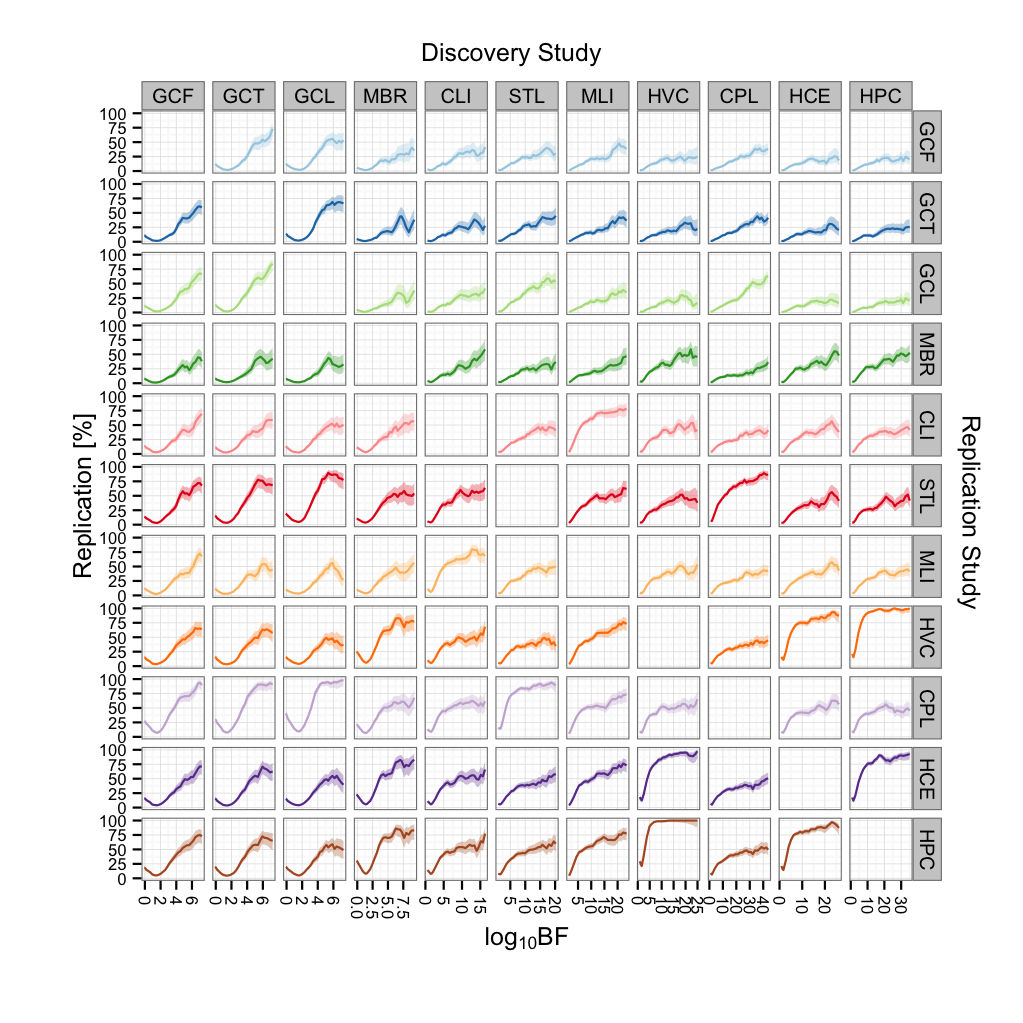

Supplement: Figure S5 — eQTL replication frequencies, by observation significance, across all cohorts. eQTL replication (y-axis), as a function of discovery significance (x-axis: ). Each column of facets depicts the set of eQTL SNPs discovered in each of 11 studies, as labeled at top. Within each column, replication frequencies are plotted separately for each replication study set, in rows and in a different color, as labeled at the right. SNPs are binned along the x-axis into 30 equally spaced intervals. Per bin replication frequencies are plotted as bold lines, 95% confidence intervals are plotted as ribbons. (TIF) [file pgen.1003649.s005.tif]

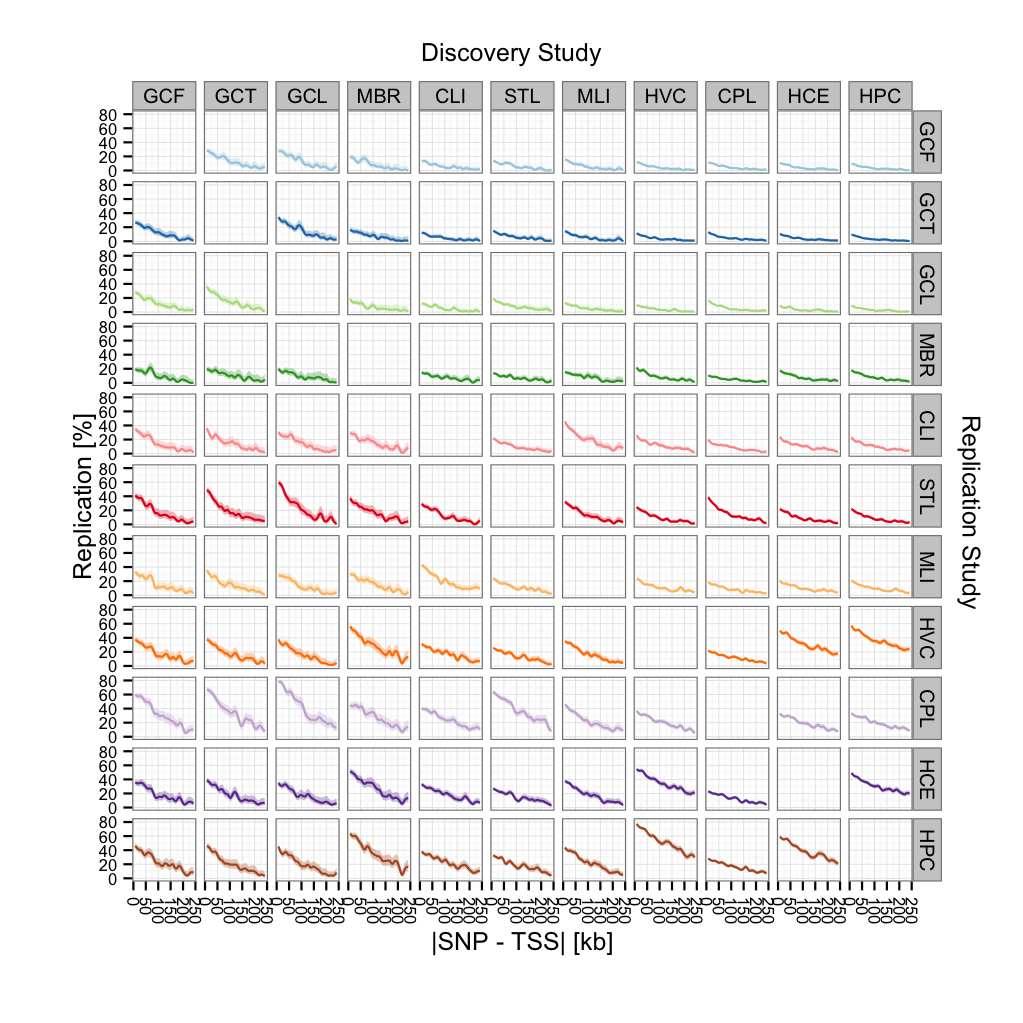

Supplement: Figure S6 — eQTL replication frequencies, by SNP position, across all cohorts. eQTL replication (y-axis), as a function of SNP position (x-axis; ). Each column of facets depicts the set of eQTL SNPs discovered in each of 11 studies, as labeled at top. Within each column, replication frequencies are plotted separately for each replication study set, in rows and in a different color, as labeled at right. SNPs are binned along the x-axis into 30 equally spaced intervals. Per bin replication frequencies are plotted as bold lines, confidence intervals are plotted as ribbons. (TIF) [file pgen.1003649.s006.tif]

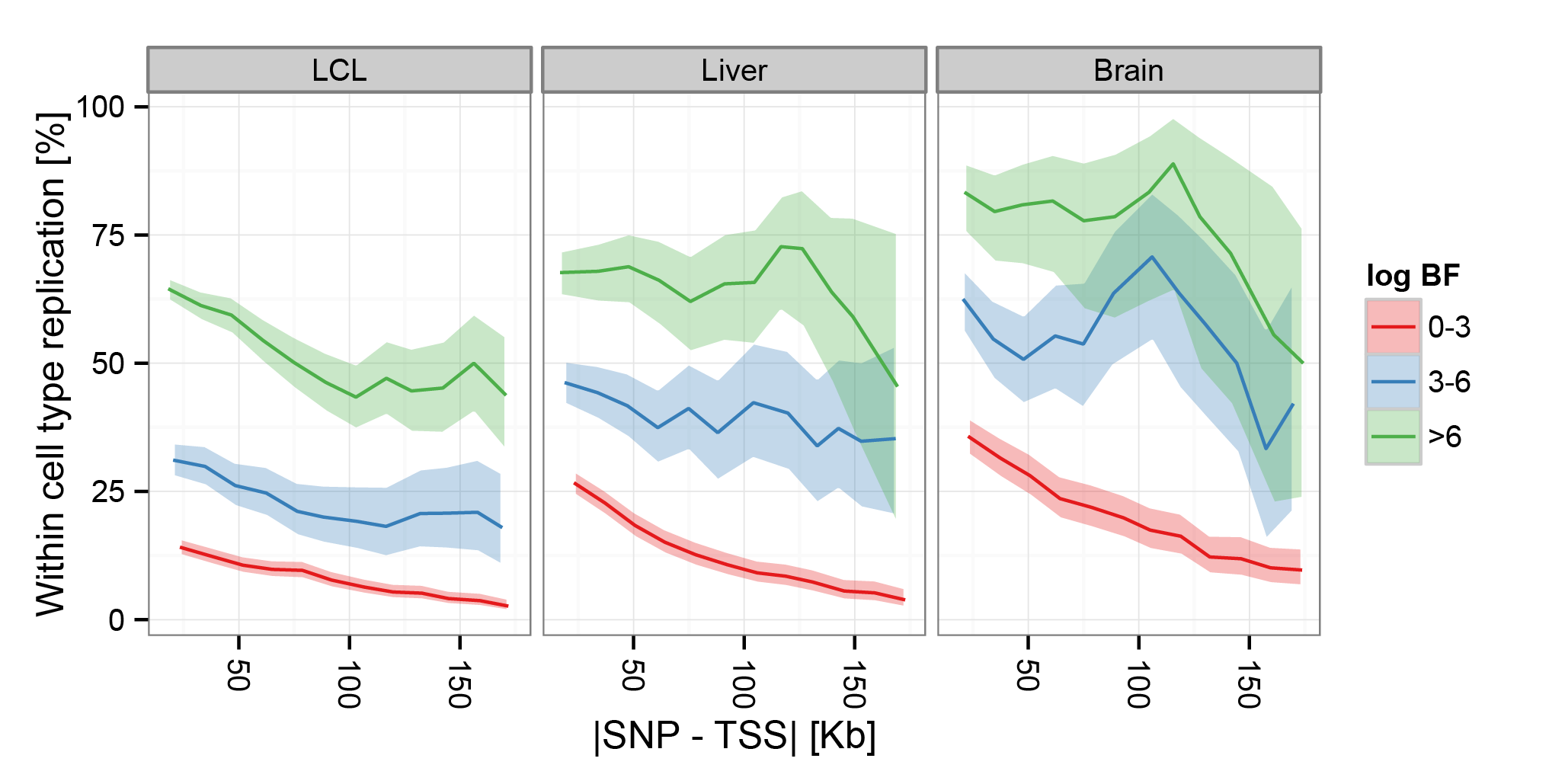

Supplement: Figure S7 — eQTL replication by SNP to TSS distance, conditioned on significance. Within cell type eQTL replication (y-axis), as a function of absolute SNP to TSS distance (x-axis). Replication frequencies are displayed separately for eQTLs with (red), (blue), and (green). SNPs have been binned along the x-axis into 15 equally spaced intervals. eQTL SNP-CRE overlaps per bin are plotted as bold lines, confidence intervals are plotted as ribbons. Each facet depicts a different study set comparison (labeled at top). (TIF) [file pgen.1003649.s007.tif]

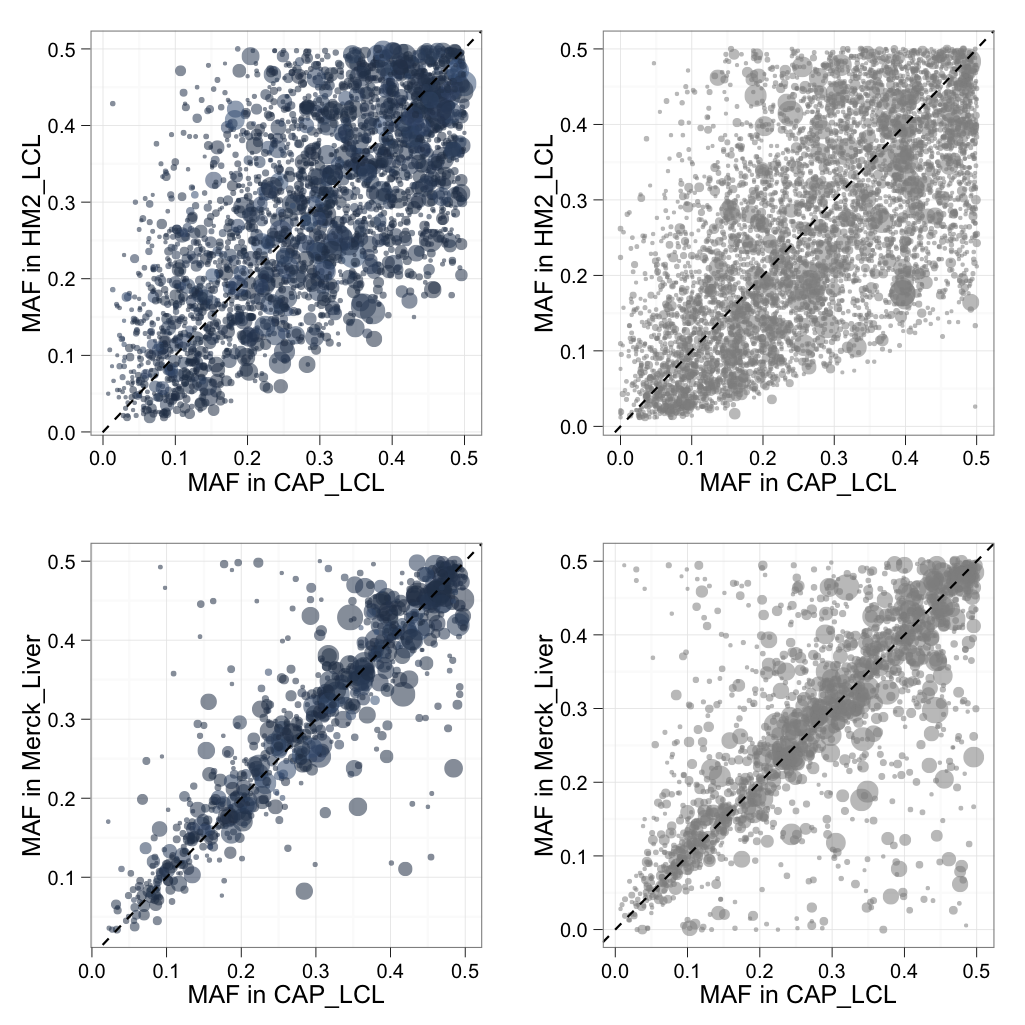

Supplement: Figure S8 — Minor allele frequency differences and replication. Minor allele frequencies in the two different populations of replicating (left column) and non-replicating (right column) eQTLs. The size of the point corresponds to the of the eQTL in CAP_LCL; the color represents the in the replication study, where blue is higher and grey (in the right-hand column) is . Top row: MAFs compared between eQTLs discovered in CAP_LCL (x-axis) that replicate (left) or fail to replicate (right) in HM2_LCL (y-axis). Bottom row: MAFs compared between eQTLs discovered in CAP_LCL (x-axis) that replicate in HM2_LCL and that replicate (left) or fail to replicate (right) in Merck_liver. (TIF) [file pgen.1003649.s008.tif]

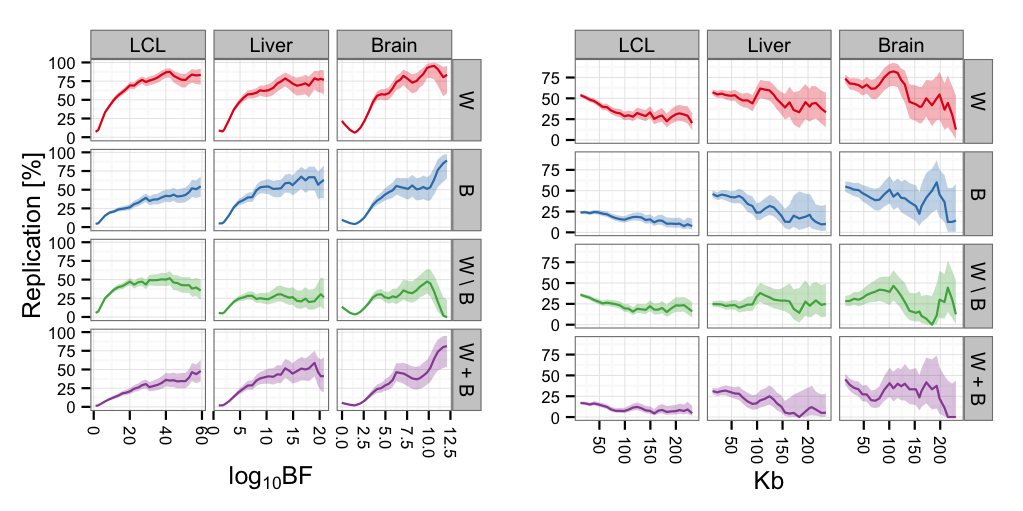

Supplement: Figure S9 — Extended eQTL replication plots. eQTL replication (y-axis), as a function of discovery significance (x-axis; left facet set) or absolute SNP to TSS distance (x-axis; right facet set; thresholded at ), as indicated at the bottom. Replication frequencies are displayed separately for within cell type (red), between cell type (blue), within but not between cell type (i.e., cell specific; green), and within and between replication (purple). SNPs have been binned along the x-axis in 30 equally spaced intervals. eQTL SNP-CRE overlaps per bin are plotted as bold lines, confidence intervals are plotted as ribbons. Each column of facets depicts a different study set comparison, as labeled at the top, for LCLs (left column), liver (middle column), and brain (right column). (TIF) [file pgen.1003649.s009.tif]

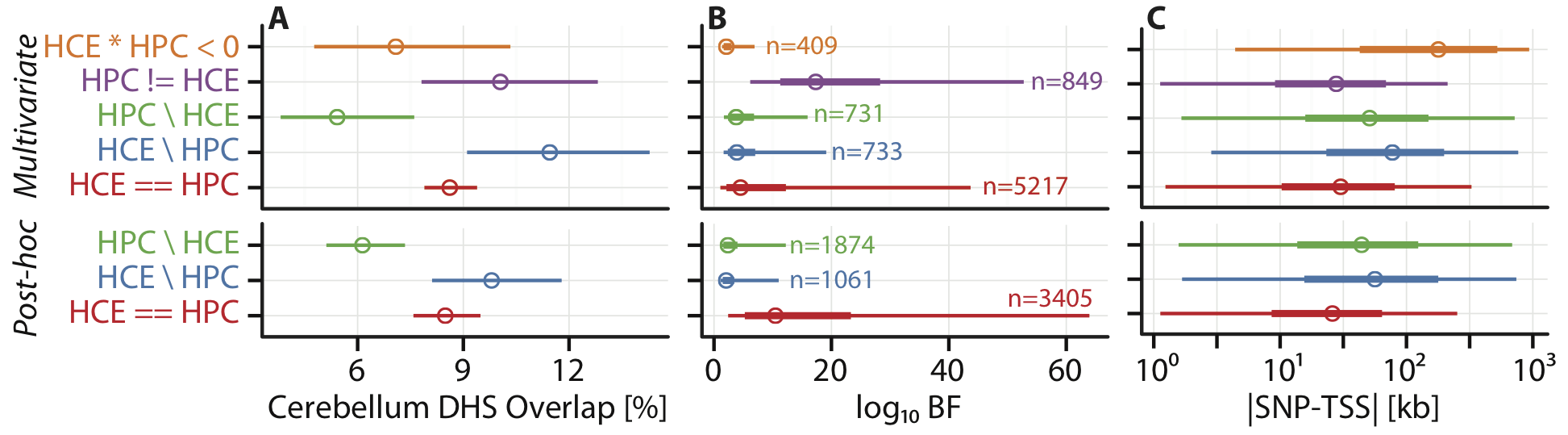

Supplement: Figure S10 — Bivariate Bayesian regression recapitulates results of post hoc comparisons. eQTL SNPs were classified by their best fitting model (denoted at left and by color). Results from bivariate cis-eQTL mapping are on top, results from post hoc models are on bottom. (A) Overlap of SNPs in each class with cerebellum DHS sites. Points denote overlap percentage, lines denote CI. (B) Distribution of (x-axis) for eQTL SNPs in each model class. Box plots denote median, inter-quartile range, and CI. (C) Distribution of absolute distances (x-axis; scale) between each SNP and its associated gene's TSS. (TIF) [file pgen.1003649.s010.tif]

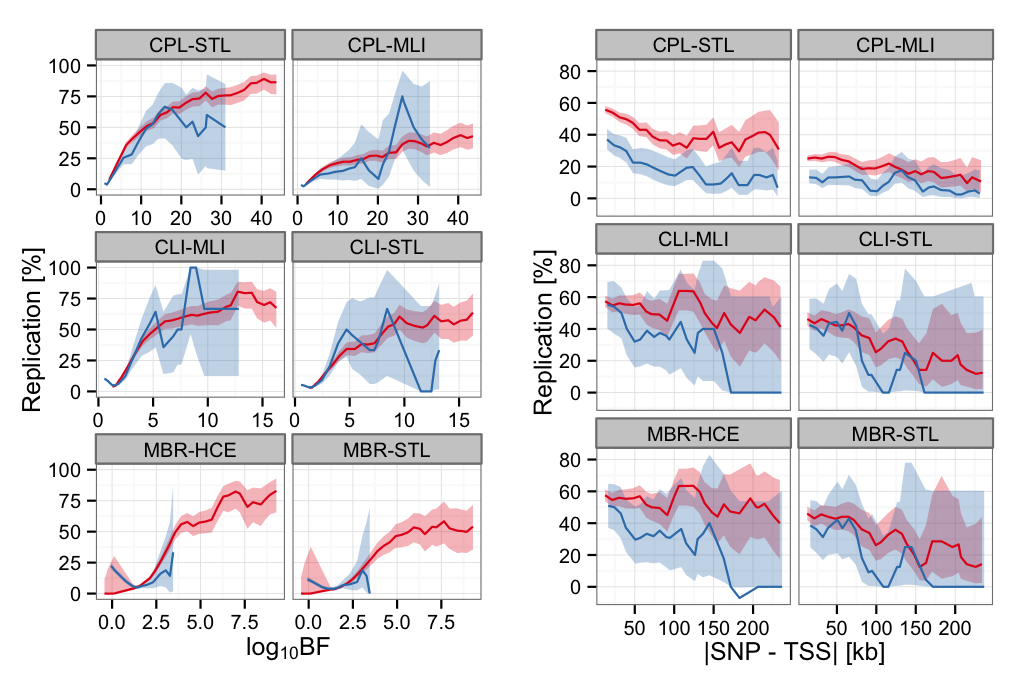

Supplement: Figure S11 — eQTL replication conditional on SNP tier. eQTL replication (y-axis), as a function of discovery significance (x-axis; ; left set of panels) or absolute SNP to TSS distance (x-axis, right set of panels). Replication frequencies are displayed separately for tier 1 (red) and tiers 2–4 (blue). SNPs have been binned along the x-axis into 30 equally spaced intervals. eQTL SNP-CRE overlaps per bin are plotted as bold lines, confidence intervals are plotted as ribbons. Each panel depicts a different study set comparison, as labeled at the top of each panel. Within cell type and between cell type replication frequencies are plotted along the left and right columns, respectively, for LCLs (top row), liver (middle row), and brain (right row). (TIF) [file pgen.1003649.s011.tif]

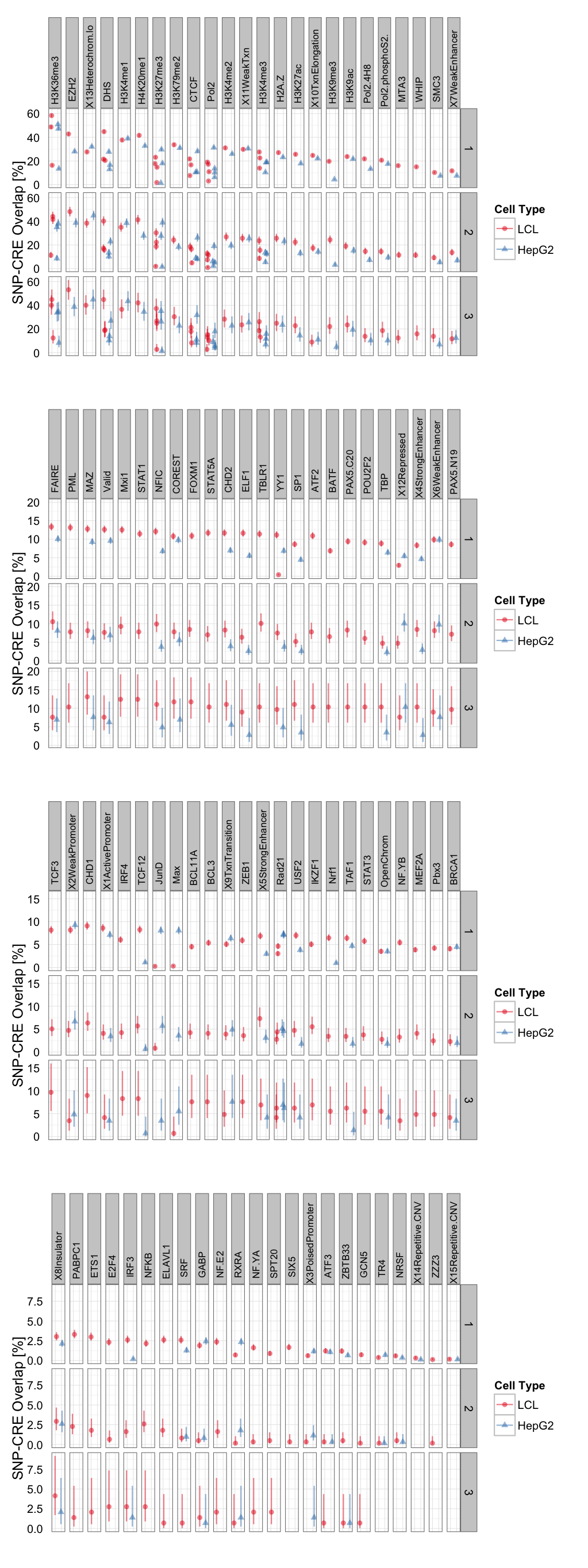

Supplement: Figure S12 — Overlap of full data set of ENCODE LCL CREs with CAP_LCL eQTLs. CAP_LCL eQTL SNP-CRE overlap (y-axis; tier means as points, confidence interval as lines), with SNPs from each tier plotted in separate panels, as indicated at right. Each CRE class is plotted in a separate panel, as labeled at top. As applicable, within each panel, overlaps are plotted separately for LCL CREs (red circles) and HepG2 CREs (blue triangles). In cases where multiple CRE data sets exist for the same CRE class (e.g., H3K27me3 marks), overlaps were calculated from each data set independently and over plotted, with a jitter. (TIF) [file pgen.1003649.s012.tif]

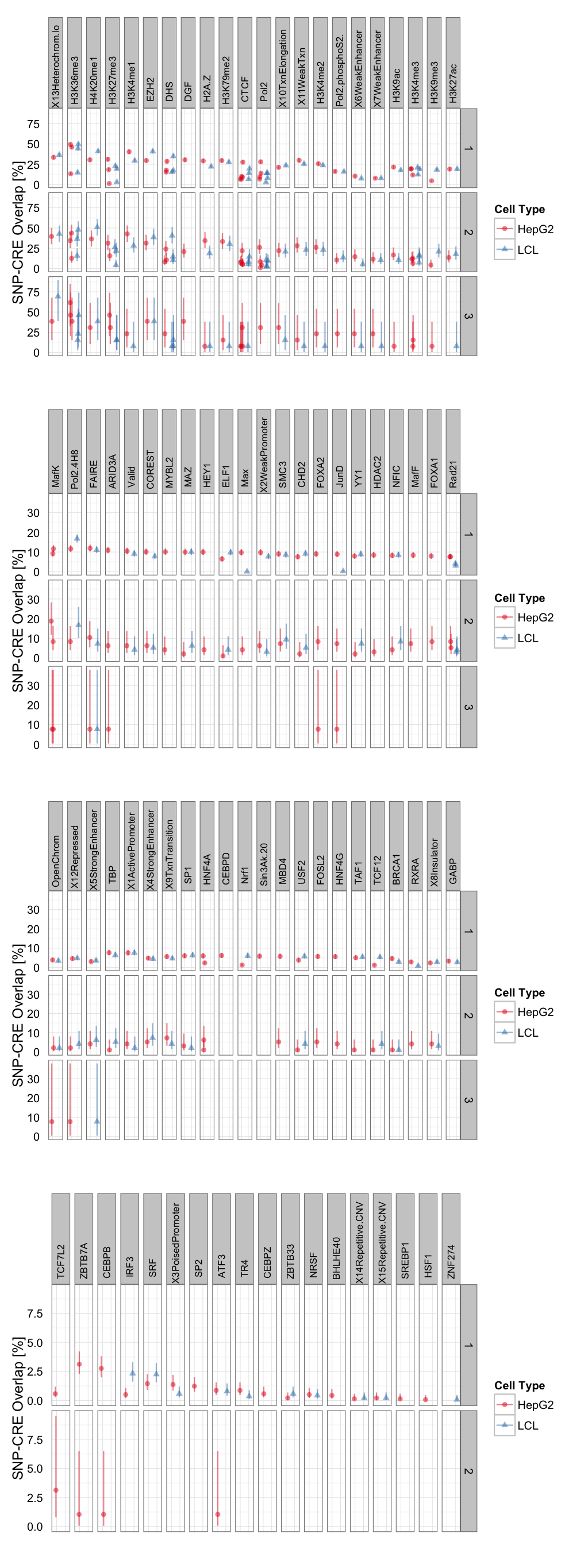

Supplement: Figure S13 — Overlap of full data set of ENCODE HepG2 CREs with UChicago_liver eQTLs. UChicago_liver eQTL SNP-CRE overlap (y-axis; tier means as points, confidence interval as lines), with SNPs from each tier plotted in separate panels, as indicated at right. Each CRE class is plotted in a separate panel, as labeled at top. As applicable, within each panel, overlaps are plotted separately for HepGe CREs (red circles) and LCL CREs (blue triangles). In cases where multiple CRE data sets exist for the same CRE class (e.g., H3K27me3 marks), overlaps were calculated from each data set independently and over plotted, with a jitter. (TIF) [file pgen.1003649.s013.tif]

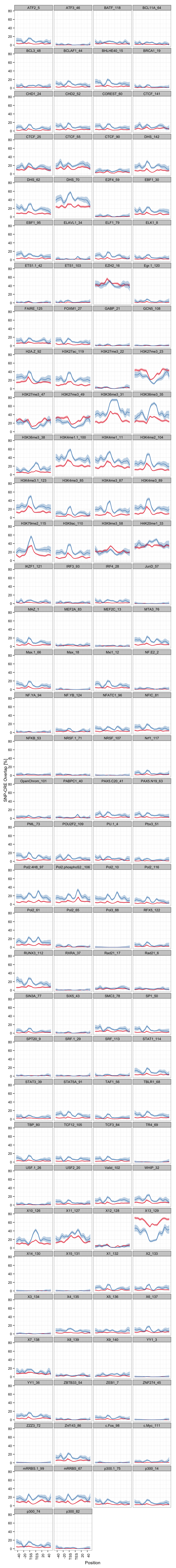

Supplement: Figure S14 — CAP_LCL eQTL associations with 142 LCL derived CRE data sets. CAP_LCL eQTL SNP () overlap with predicted cis-regulatory elements. Each panel depicts overlap with distinct CRE data sets, as labeled at top. In each panel, SNPs are grouped into 25 equally spaced bins within the 50 kb upstream and downstream of the TSS and TES, and 10 bins between the TSS and TES. Each bin is plotted along the x-axis. Bold lines depict the percentage, per bin, of SNPs overlapping the CRE class, ribbons depict confidence interval. Observed eQTL SNPs are plotted in blue and randomly drawn cis-linked SNPs at expressed genes in red. (TIF) [file pgen.1003649.s014.tif]

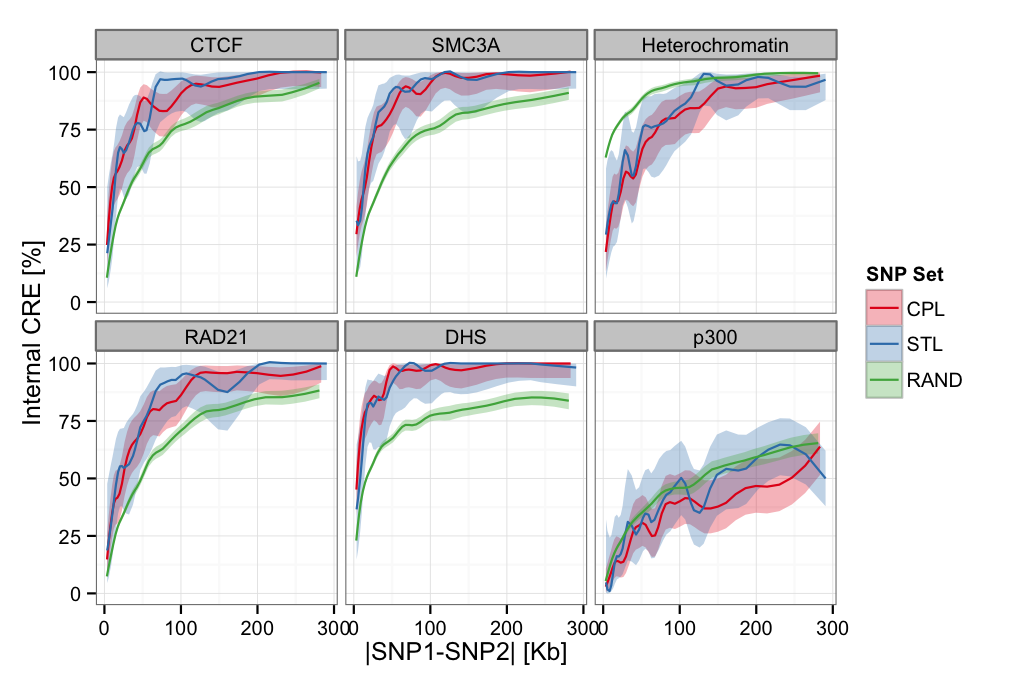

Supplement: Figure S15 — CTCF binding sites are enriched between SNPs independently associated with the same gene expression trait. Percentage of primary and secondary CAP_LCL (red) and Stranger_LCL (blue) LCL eQTL SNP pairs that have an intervening CRE (y-axis; SNP pairs were binned by the distance between them, bold lines depict bin frequency, ribbons depict confidence interval) as a function of the absolute distance between the SNPs (x-axis). Randomly drawn cis-linked SNPs are displayed in green. Each panel depicts the analysis of a different CRE data set, as labeled at top, including CTCF, SMC3, and Rad21, which have each been shown to mark enhancer blocking insulators [73], DHS sites, which promiscuously mark insulators and other CRE classes, chromHMM defined heterochromatin regions, and p300 sites. (TIF) [file pgen.1003649.s015.tif]

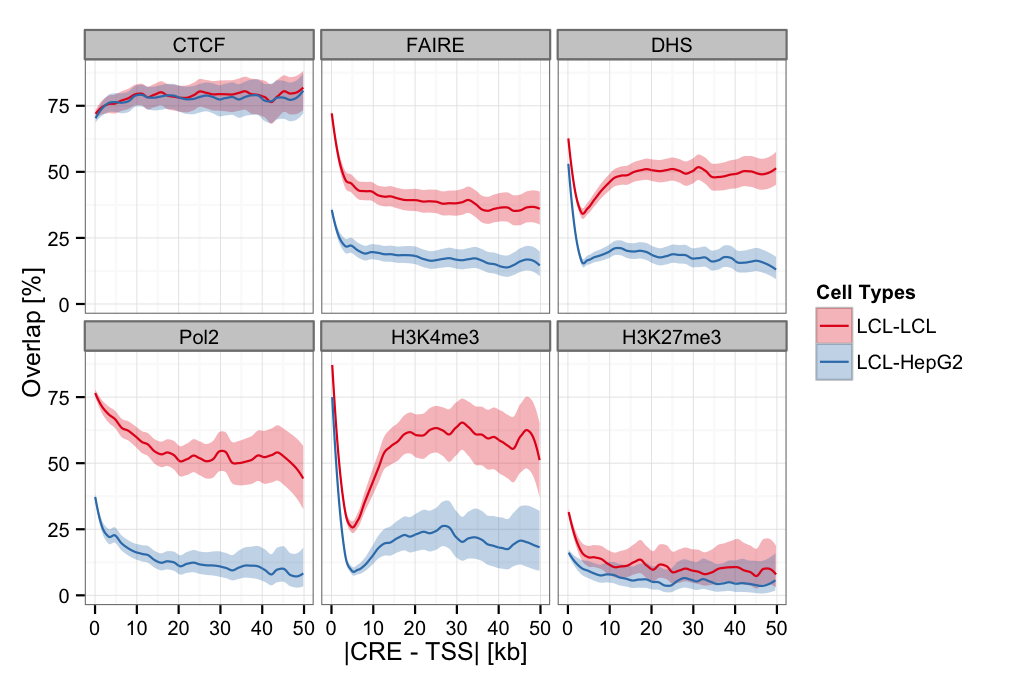

Supplement: Figure S16 — Overlap of ENCODE data sets across cell types. Overlap between CRE data sets (y-axis), as a function of absolute CRE to TSS distance (x-axis). Data are plotted separately for comparisons between two different LCL lines (red; e.g., between GM12878 and GM060990) and between LCLs (GM12878) and HepG2 cells (blue). CREs are binned into 30 equally spaced intervals along the x-axis. Per bin CRE overlaps are plotted as bold lines, confidence intervals are plotted as ribbons. Data are plotted separately for each of six different CRE types, as labeled at the top of each panel. Note the striking difference between the cell specificity of CTCF binding sites and each other CRE class. (TIF) [file pgen.1003649.s016.tif]

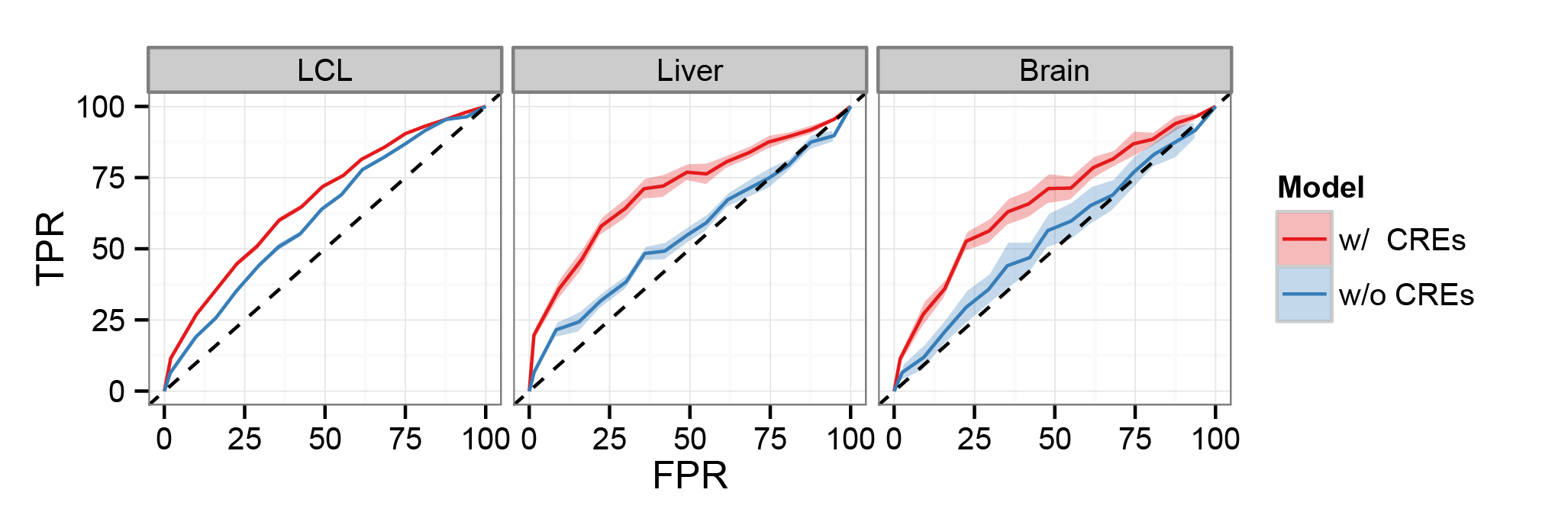

Supplement: Figure S17 — CRE data improve accuracy of cell specific eQTL prediction. ROC curves depicting the performance of random forest classifiers to predict within cell type specific reproducibility, either trained with CRE data (red) or without CRE data (blue). Facets depict predictions for LCL, liver, and brain eQTL SNPs (labeled at top). True positive rates (y-axis) and false positive rates (x-axis) were quantified by tenfold cross validation. AUCs from models with and without CRE training, respectively, were 0.67 and 0.61 (LCL), 0.71 and 0.57 (liver), and 0.68 and 0.57 (brain). (TIF) [file pgen.1003649.s017.tif]

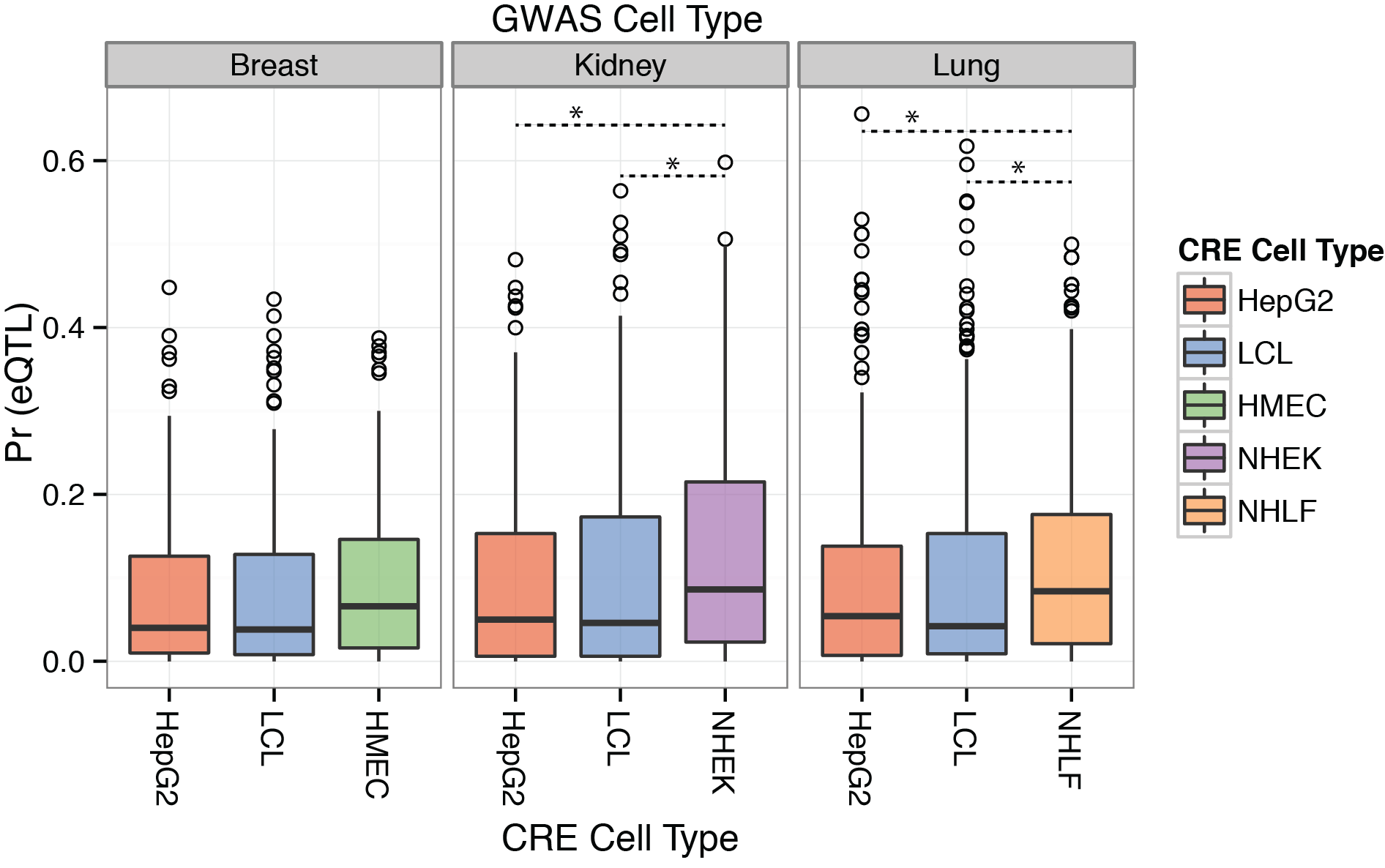

Supplement: Figure S18 — Regulatory element overlap predicts GWAS SNP function. Boxplot of the distribution of random forest classifier predictions. A classifier was trained to discriminate LCL and liver eQTL SNPs from non-eQTL SNPs on the basis of SNP overlap with CREs from LCLs and HepG2 cells. The trained classifier was then applied to SNPs associated with phenotypes of relevance to breast, kidney, and lung function (facets labeled at top). The probability that each SNP is an eQTL (y-axis, as box plot) was calculated using CREs from matched and unmatched cell types (listed on the x-axis and color coded). Asterisks denote significant differences in probability distributions (Wilcoxon signed ranks test, ). (TIF) [file pgen.1003649.s018.tif]

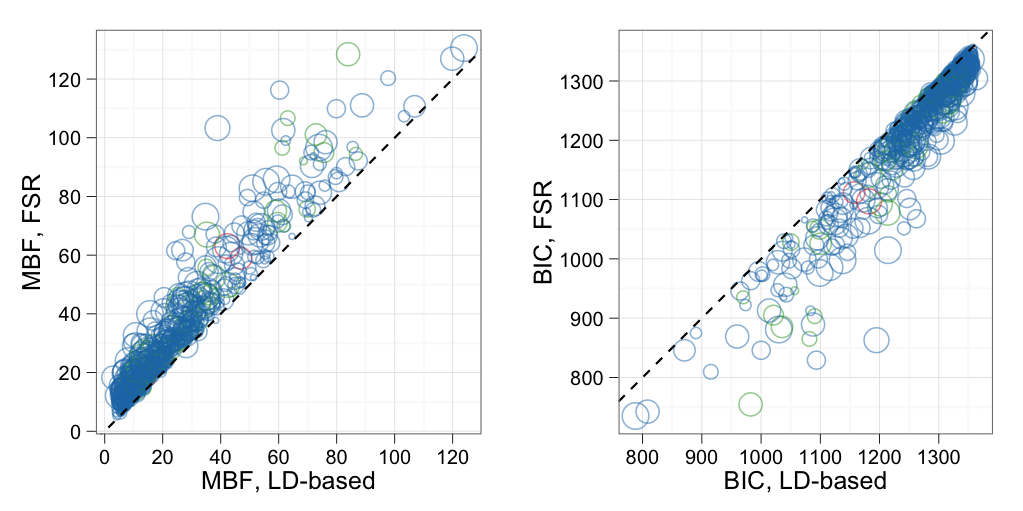

Supplement: Figure S19 — Quantification of allelic heterogeneity by forward stepwise regression and an LD-based method. Forward stepwise regression was applied to genes with allelic heterogeneity as identified by the LD-based method described in the text. For each gene, the resulting pair of models are contrasted as follows. At left, multivariate of the FSR model (y-axis) is plotted as function of the multivariate of the LD based model (x-axis). At right, the Bayesian information criterion (BIC) of the FSR model (y-axis) is plotted as function of the BIC of the LD based model (x-axis). Circle size and color depicts the number of SNPs identified by the FSR and LD-based models, respectively (blue = 2 SNPs, green = 3 SNPs, red = 4 SNPs). We note that, in one gene, NINJ1, FSR found fewer independent eQTLs than our LD-based method; interestingly, the tertiary eQTL SNP for this gene had a lower univariate than the conditional ( versus ), implying a possible SNP-SNP interaction. For genes () the two methods found the same numbers of independent eQTL SNPs, and for genes () FSR identified additional eQTL SNPs (Table S9). (TIF) [file pgen.1003649.s019.tif]
